# Novel role for alpha-2-macroglobulin (A2M) as a disease modifying protein in senile osteoporosis

**DOI:** 10.3389/fcell.2023.1294438

**Published:** 2023-10-30

**Authors:** Siddaraju V. Boregowda, Christopher L. Haga, Valentina M. Supper, Cori N. Booker, Donald G. Phinney

**Affiliations:** Department of Molecular Medicine, The Herbert Wertheim UF Scripps Institute for Biomedical Innovation and Technology, Jupiter, FL, United States

**Keywords:** osteoporosis, skeletal stem cells, mesenchymal stromal cells, A2M, aging

## Abstract

**Introduction:** In the rapidly aging U.S. population, age-induced bone loss (senile osteoporosis) represents a major public health concern that is associated with a significant increased risk for low trauma fragility fractures, which are debilitating to patients, cause significant morbidity and mortality, and are costly to treat and manage. While various treatments exist to slow bone loss in osteoporosis patients, these suffer from poor tolerability and label restrictions that limit their overall effectiveness. Over the past decade, skeletal stem/progenitor cells (SSPCs), which are the main precursor of osteoblasts and adipocytes in adult bone marrow (BM), have emerged as important players in osteoporosis.

**Methods:** Age-induced skeletal pathology was quantified in elderly (24-month-old) vs. mature (3-month-old) mice by micro-CT and changes in SSPC abundance in the BM of these mice was quantified by fluorescence-activated cell sorting (FACS). SSPCs from elderly vs. mature mice were also analyzed by RNA-Seq to identify differentially expressed genes (DEGs), and gain and loss-of-function studies were performed in human BM-derived mesenchymal stromal cells (BM-MSCs) to assess A2M function.

**Results:** Elderly mice were shown to exhibit significant age-induced skeletal pathology, which correlated with a significant increase in SSPC abundance in BM. RNA-seq analysis identified alpha-2-macroglobulin (A2M), a pan-protease inhibitor that also binds inflammatory cytokines, as one of the most downregulated transcripts in SSPCs isolated from the BM of elderly vs. mature mice, and silencing of A2M expression in human BM-MSCs induced their proliferation and skewed their lineage bifurcation toward adipogenesis at the expense of osteogenesis thereby recapitulating critical aspects of age-induced stem cell dysfunction.

**Conclusion:** These findings identify A2M as a novel disease modifying protein in osteoporosis, downregulation of which in bone marrow promotes SSPC dysfunction and imbalances in skeletal homeostasis.

## 1 Introduction

Currently, over 10 million adults in the United States age 50 or older have osteoporosis and another 33 million suffer from osteopenia (Golden et al., 2009; Wright et al., 2014), a serious osteoporosis risk factor, making osteoporosis one of the most common chronic age-associated disorders in humans (Burge et al., 2007; Jeremiah et al., 2015). Characterized by significant decreases in bone mass, denisty and rigidity, osteoporosis patients have a significant increased risk for low-trauma fragility fractures and their complications (Kim et al., 2010; Jiao et al., 2015). For example, it is estimated that ∼50% of women and ∼25% of men age 50 or older will experience at least one osteoporosis-related bone fracture, which are associated with significant morbidity and mortality and are costly to treat and manage (Burge et al., 2007; Tu et al., 2018). Numerous studies have identified imbalances in the activity of bone forming osteoblasts, bone resorbing osteoclasts and mechanosensitive osteocytes as the main driver of bone loss in osteoporosis (Baron and Kneissel, 2013), and while various treatments that decrease bone loss (Mosekilde et al., 2000; Russell, 2011; Siu et al., 2013; Plotkin et al., 2015) or stimulate new bone formation (Cosman et al., 2017; Markham, 2019) exist, their widespread use is limited by poor tolerability in some patients and adverse side effects associated with long-term use.

Increased marrow adipose tissue (MAT) volume is recognized as a prominent feature of osteoporosis, which accelerates bone loss due to the anti-osteogenic activity of secreted fatty acids, adipokines and RANKL (Meunier et al., 1971; Rosen and Bouxsein, 2006; Botolin and McCabe, 2007; Zhang et al., 2011; Sadie-Van Gijsen et al., 2013; Devlin and Rosen, 2015). This observation has implicated skeletal stem/progenitor cells (SSPCs), which serve as the main precursor of osteoblasts and adipocytes in adult bone marrow (BM), as important players in osteoporosis (Zhou et al., 2014; Panes et al., 2016; Yue et al., 2016). While several distinct cell populations that ostensibly function as SSPCs in BM have been identified (Cao et al., 2020), several independent studies have implicated the Lin^−^LEPR^+^ cell fraction, which encompasses SSPCs and osteogenic and adipogenic progenitors, in skeletal pathophysiology. First, Yue et al. (2016), demonstrated that targeted deletion of Lin^−^LEPR^+^ cells in BM protects mice from bone loss and MAT accumulation in response to high fat diet feeding and Deng et al. (2021), showed that targeted deletion of the histone lysine demethylase KDM4B in the *LepR* cell compartment exacerbates age-induced bone loss and MAT accumulation by inducing stem cell exhaustion. Most recently, Shen et al. (2021), reported that a subpopulation of osteolectin (OLN) expressing Lin^−^LEPR^+^ cells decline precipitously with aging in mice. These findings are consistent with other studies linking age-related bone loss to decreased proliferation, increased senescence, and impaired osteogenesis of bone marrow-derived mesenchymal stem cells (BM-MSCs) (Bellantuono et al., 2009; Kim et al., 2012), which are culture-expanded populations that retain SSPC properties. Together, these studies implicate SSPC dysfunction as a driver of skeletal pathology in senile osteoporosis.

Herein, we demonstrate that size of the Lin^−^LEPR^+^ pool in BM is expanded in elderly (24-month-old) vs. mature (3-month-old) mice even though elderly mice showed significant bone loss based on micro-CT analysis. To better contextualize this result, we performed RNA sequencing-based transcript profiling (RNA-seq) of Lin^−^LEPR^+^ SSPCs from these mice, which identified alpha-2-macroglobulin (A2M) as a highly downregulated transcript in response to aging. A2M gain and loss of function studies further demonstrated that increased proteolytic activity augmented proliferation and skewed bifurcation of BM-MSCs toward adipogenesis at the expense of osteogenesis thereby recapitulating aspects of age-induced stem cell dysfunction *in vivo*. These data, together with studies showing that A2M plasma levels and BM-MSC A2M promoter methylation levels are negatively and positively correlated, respectively, with age in humans (Birkenmeier et al., 2003) and that genetic deletion of *A2m* results in deleterious skeletal alterations in mature mice, identify A2M as a putative disease modifying protein in senile osteoporosis.

## 2 Materials and methods

### 2.1 Mice

All studies involving mice were reviewed and approved by the Institutional Animal Care and Use Committee at the Herbert Wertheim UF Scripps Institute for Biomedical Innovation and Technology. C57Bl/6 male mice (Jackson labs) were maintained in ventilated cages on a 12 h light:dark cycle and fed standard chow for the duration of the study unless indicated otherwise. Mice purchased at 18 months old were housed until they reached 24 months of age. Mice used in this study were euthanized by asphyxiation using a time controlled Euthanex device that delivers CO_2_ gas at a flow rate of 10%–30% of the container volume per minute. This method is consistent with recommendations from the AVMA guidelines for euthanasia of animals.

### 2.2 Cell isolation and culture

Human BM-MSCs were isolated from aspirates of the iliac crest (Lonza, United States) as previously described (Boregowda et al., 2016) and cultured in complete culture medium (CCM) consisting of α-MEM supplemented with 100 U/mL Penicillin, 100 μg/mL Streptomycin, 2 mM L-glutamine and 17% FBS (Atlanta Biologicals, Inc.) at 37°C in 5% CO_2_ in a humidified chamber. Media changes were performed every 3-4d and BM-MSCs were harvested at ∼70% confluency with 0.25% trypsin-EDTA. Media and supplements were purchased from Gibco unless indicated otherwise. Lin^−^LEPR^+^ and Lin^−^LRP1^+^ cells were enriched from BM using a published method with modifications (Zhou et al., 2014). Briefly, BM pooled from femurs/tibiae of mice was digested for 15 min at 37°C with 500 μg/mL Liberase DL (Sigma-Aldrich) and 400 μg/mL DNAseI (Sigma-Aldrich). The digestion was repeated a total of three times, and after each digestion the single cell suspension was transferred to a fresh vial on ice and the remaining undigested marrow was added with fresh digestion buffer. Liberated cells were pooled and suspended in HBSS, stained with CD16/32 Fc block (BD, #553142, 1:40), FITC anti-Ter119 (Tonbo #35-5921, 1:100), FITC anti-CD31 (BD, #553372, 1:50), FITC anti-CD45 (BD, #553078, 1:100), biotinylated anti-LEPR (R&D, #BAF497, 1:33), biotinylated anti-LRP1 (Novus Biologicals, #NB100-64808B, 1:33), donkey-anti-goat Alexa 647 (Invitrogen, #A21447, 1:40) and/or Streptavidin BV-421 (BD Biosciences, #563259) antibodies and propidium iodide (PI) (BD, #51-66211E, 1:100) and the subpopulations of interest isolated by FACS using a BD FACS Aria at a flow rate of <10,000 cells/sec and a 100 μM nozzle.

### 2.3 Micro-CT

Tibiae were carefully removed at the knee, keeping the proximal tibia and muscles intact, stored in 10% neutral buffered formalin, and shipped to the University of Toledo for analysis. Tibiae were dissected, fixed overnight in 4% paraformaldehyde, and stored in 70% ethanol at 4°C. Bone and MAT scans were conducted using a µCT35 micro-CT system (SCANCO Medical AG) with the x-ray source operating at 70 kVp and 114 μA energy settings, and recording 500 projections/180° acquired at 300 ms integration time using. Scans of trabecular bone in the proximal tibia consisted of 300 slices covering 2.1 mm from the growth plate, and scans of cortical bone consisted of 57 slices covering 0.4 mm of the tibia midshaft. Segmentation of trabecular bone images was conducted on 200 total slices beginning 10 slices from the growth plate following manual contouring (optimized gray-scale threshold of 220 per mille, equivalent to 3313 Hounsfield units, or μ of 1.76). Segmentation of cortical bone images are conducted on the entire image stack of 57 slices following semi-automatic contouring (260 per mille threshold, 3673 Hounsfield Units). Trabecular and cortical bone morphometric parameters were calculated directly from voxel values with a 7 μm nominal resolution voxel for trabecular bone and 12 μm for cortical bone. Lipid distribution and volume was conducted on decalcified bone specimens stained for 2 h in 0.1 M sodium cacodylate buffer (pH 7.4) with 2% osmium tetroxide. Images of lipid deposits were acquired at 12 µm resolution and quantified directly from voxel volumes from whole tibiae. The data shown from bone and MAT scans was generated using Evaluation Program V6.5-3 (Scanco Medical AG) and analyzed using recommended guidelines (Bouxsein et al., 2010).

### 2.4 Metabolic profiling

Blood was drawn via cardiac puncture after euthanasia and collected in uncoated 1.5 mL tubes. After clot formation, the tubes were centrifuged 5 min at 1,000 g, the serum was decanted and stored at −80°C. Leptin levels were quantified using the Quantikine Mouse/Rat Leptin ELISA Kit (R&D Systems MOB00).

### 2.5 Cell proliferation and differentiation

BM-MSCs were stained with 10 µM carboxyfluorescein succinimidyl ester (CSFE, Invitrogen) at 37°C for 10 min, quenched with five volumes of CCM, washed 3x with PBS, collected by centrifugation at 500 g for 10 min, then plated at 1,000 cells/cm^2^ and cultured for 7 days at 37°C with media changes every 2–3 days prior to flow cytometric analysis. Where indicated, BM-MSCs were transfected with a scrambled or *A2m-*specific siRNA (Ambion A2M silencer Select, #4392420) using the reverse transfection method as described previously (Boregowda et al., 2016). Additionally, BM-MSCs (∼3,000 cells/cm^2^) were incubated in adipogenic induction media (AIM; CCM supplemented with 500 nM Dexamethasone, 500 µM Isobutylmethylxanthine, 500 µM Indomethacin, 1 U Penicillin and 1 μg/mL streptomycin) or osteogenic induction media (OIM; DMEM low glucose supplemented with 10% FBS, 100 nM Dexamethasone, 10 µM β-glycerol-phosphate, 147.5 μM L-Ascorbic acid 2-phosphate, 1 U Penicillin and 1 μg/mL streptomycin) for 10 or 14 days, respectively, with media changes every 2–3 days. To measure adipogenesis, cells were incubated for 10 min at 37°C in AdipoRed reagent and the extent of staining quantified spectroscopically (485 nm). For osteogenesis, cells were incubated in 10% neutral buffered formalin for 1 h, washed with de-ionized water and stained with Alizarin Red S (pH = 4.2) for 20 min at room temperature. Monolayers were then rinsed with de-ionized water until clear, washed with PBS, and extracted with 10% (w/v) cetylpyridinium chloride in 10 mM sodium phosphate, pH 7.0 for 15 min at room temperature and extracted dye quantified spectroscopically (562 nm). For gain and loss-of-function experiments, BM-MSCs from two donor populations (RD02, RD05) were plated 48-well plates (∼20,000 cells/well) and after 1-3d the media (0.5 mL/well) was replaced with AIM or OIM supplemented with 10 μL of PBS or Trypsin (2.5%). Media was replaced every 7d and cultures terminated after 10 or 21d, respectively, and cell monolayers stained as described above. Alternatively, BM-MSCs were cultured in AIM or OIM alone or supplemented with 0.5 mg recombinant human A2M (rhA2M)/mL (R&D Systems, #1938-PI), cultured for a total of 7 or 5 days, and then stained as above. Spectroscopic analyses were performed using a SpectraMax^®^ M5e Multi-Mode Microplate Reader (Molecular Devices, LLC.) and images were acquired using a Leica DMI3000B upright fluorescent microscope attached to a DFC295 digital camera (Micro Optics of Florida, Inc.). All experiments included non-stimulated cells incubated for the same duration as controls.

### 2.6 RNA sequencing

Total RNA was pooled from 3 or 4 mice per group to increase yields for library prep. RNA was run on an Agilent 2100 Bioanalyzer RNA pico chip (Agilent Technologies 5067-1513) for quality assessment and quantification and subsequently concentrated with a SpeedVac Vacuum Concentrator (Thermo Scientific). Libraries were prepared in an RNase-free working environment. For all samples except 14wk AMB and HU MSC (processed as total RNA), poly-adenylated RNAs were selectively isolated from total RNA (∼10 ng) using poly-T oligos attached to magnetic beads according to the manufacturer’s guidelines in the NEBNext poly(A) mRNA magnetic isolation module (NEB E7490). Library preparation from enriched mRNA and total RNA was completed with the NEBNext Ultra II Directional RNA kit (NEB E7760). Briefly, the RNA samples were chemically fragmented in a buffer containing divalent cations and heating at 94°C for 15 min. The fragmented RNA was random hexamer primed and reverse transcribed to generate the first strand cDNA. The second strand was synthesized after removing the RNA template and incorporating dUTP in place of dTTP. Incorporation of dUTP quenched the second strand during PCR amplification and therefore the strand information was preserved. The double-stranded (ds) cDNA was purified with 2.0X Agencourt AMPure XP bead (Beckman Coulter A63881) ratio to retain insert fragments >80 nt. The ds cDNA was end repaired and adenylated at the 3’ end. A corresponding “T” nucleotide on the adaptors was utilized for ligating the adaptor sequences to the double stranded cDNA. The adaptor ligated DNA was purified using 1.2X bead ratio and PCR amplified using 15 cycles to incorporate a unique barcode and to generate the final libraries. The final libraries were size selected and purified using 1.0X AMPure XP beads to remove any primer dimers and validated on a Bioanalyzer High Sensitivity DNA chip and normalized to 1 nM. After equimolar pooling, the libraries were loaded at a 1.8 pM concentration, and sequenced on a NextSeq 500 (Illumina) with 2 bp × 40 bp paired-end chemistry. On average, 20–25 million pass filter (base quality score >Q30 suggesting less than 1 error in 1,000 bp) reads were generated.

### 2.7 RNA-seq data analysis

All raw RNA-Seq data processing was conducted using the Galaxy web application (Afgan et al., 2018). Collected reads were downloaded as raw FASTQ files and quality checks were performed using the FASTQC application (Andrews, 2010). CutAdapt (Martin, 2011) was used to remove adapter sequences and short reads. Reads were mapped against the NCBI Build 37 mm9 mouse reference genome using the HISAT2 (Kim et al., 2019) and featureCounts (Liao et al., 2014 April 1) application packages. Raw read counts were then exported for downstream analysis in the R software package environment. Differential gene expression analysis was carried out in R using the edgeR package (Robinson et al., 2009). Low expressing raw reads across samples were filtered out such that the sum of counts was greater than 2 across any given transcript. Raw read counts were normalized with the edgeR calcNormFactors function to generate a model of normalized reads (cpm) which were then filtered to only include protein coding transcripts in subsequent analyses. A dispersion value of 0.2 was used for differential expression exact test analysis. Differentially expressed genes (DEGs) were used to create ranked ordered lists for gene ontology (GO), gene set enrichment analysis (GSEA), and KEGG pathway analysis using the clusterProfiler R package (Yu et al., 2012). PCA analysis was carried out using the DESeq2 software package (Love et al., 2014). Heatmaps were generated using the Pheatmaps R package (Kolde, 2019). Heatmaps are displayed as a calculated Z score across each row of transcripts and display the top 1000 most highly variable transcripts. The Enhanced Volcano package (Blighe et al., 2019) was used to generate volcano plots of DEGs.

### 2.8 Western blotting

BM-MSCs were washed 2x with ice-cold PBS and collected in RIPA buffer (50 mM Tris-Cl ph7.4, 150 mM NaCl, 1% NP-40, 0.5% sodium deoxycholate, 0.1% sodium dodecyl sulfate) by gently scraping. Lysates were cleared by centrifugation at 12,000 g for 20 min and protein content measured using the Bradford method (Bio-Rad). Protein samples were suspended in 4X NuPAGE LSD sample buffer, heated for 3 min at 95°C, and separated by electrophoresis on NuPAGE 4%–12% Bis-Tris polyacrylamide gels (Invitrogen). Proteins were transferred to PVDF membranes, incubated with TBST (10 mM Tris, pH 8.0, 150 mM NaCl, 0.1% Tween 20) and 5% non-fat dry milk (Bio-Rad) for 1 h at room temperature, rinsed in TBST and incubated overnight at 4°C in TBST containing 5% BSA with the following antibodies; mouse anti-hGAPDH (SantaCruz, #sc-47724), rabbit anti-hLRP1(Cell Signaling, #64099S) and rabbit anti-hA2M (Cell Signaling, #71610S) antibodies followed by IRDye^®^ 680LT Donkey anti-Mouse IgG Secondary Antibody (red, Licor #926-68022) and IRDye^®^ 800CW Donkey anti-Rabbit IgG Secondary Antibody (green, Licor #926-32213). Proteins were visualized using an Licor Odyssey Imaging System and both colors were imaged using a single scan.

### 2.9 Statistical analysis

The statistical significance between two independent experimental groups was assessed using a two-tailed, unpaired Student’s t-test. The statistical significance among more than two groups was assessed using a one-way ANOVA with multiple comparisons assessed using the Tukey’s test. Significance level was set at *p* ≤ 0.05. All data represent mean ± standard deviation.

## 3 Results

### 3.1 Elderly mice exhibit skeletal pathology consist with senile osteoporosis

**FIGURE 1:**
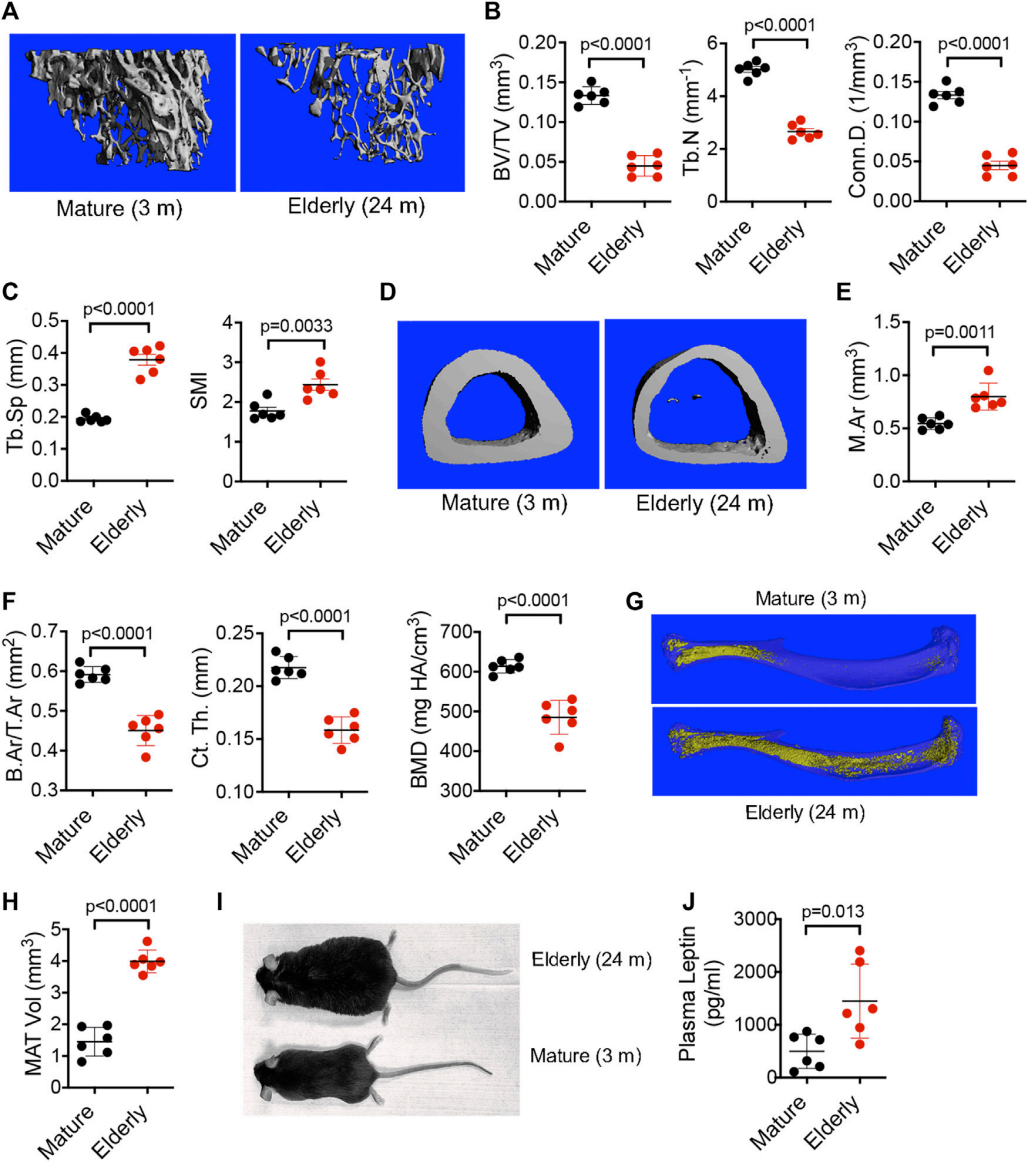
Aging promotes bone loss and MAT expansion. **(A,D)** Representative micro-CT images of the proximal **(A)** and midshaft **(D)** tibiae of mature (3-month-old) and elderly (24-month-old) male C57Bl/6 mice. **(B,C)** Quantification of BV/TV, Tb.N, and Conn. D **(B)** and Tb.Sp and SMI **(C)** by micro-CT in mice from **(A)**. (**E,F)** Quantification of M.Ar **(E)** and B.Ar/T.Ar, Ct. Th, and BMD **(F)** by micro-CT in mice from **(D)**. **(G)** Representative micro-CT images of MAT in tibiae stained with osmium tetroxide from mature (3-month-old) and elderly (24-month-old) male C57Bl/6 mice. **(H,J)** Quantitation of MAT volume **(H)** and plasma leptin levels **(J)** in mice from **(G)**. **(I)**, Photograph of a 3-month-old (mature) and 24-month-old (elderly) male C57Bl/6 mouse. Data are mean ± SD (*n* = 6 mice/group) and *p*-values are by paired Student’s t-test.

To interrogate the role of BM resident SSPCs in senile osteoporosis, we initially quantified age-induced skeletal pathology in male mice by micro-CT. Although the exact peak age of development is not rationally established, 3-month-old mice are recognized as equivalent to fully mature adult humans (20–30 years), 18-month-old mice are representative of middle aged (38–56 years) humans, and 24-month-old mice are representative of elderly (>69 years) humans (Dutta and Sengupta, 2016). Analysis of the proximal tibiae (Figure 1A) of mature (3-month-old) vs. elderly (24-month-old) male mice revealed significant decreases in bone volume as a percentage of total volume (BV/TV), trabecular number (Tb.N), and connectivity density (Conn.D) (Figure 1B) and significant increases in trabecular spacing (Tb.Sp) and structure model index (SMI) (Figure 1C) in response to aging. Analysis of the tibiae midshaft (Figure 1D) further revealed that elderly vs. young mice exhibited a significant increase in total marrow area (M.Ar) (Figure 1E) and significant decreases in bone area as a fraction of total area (B.Ar/T.Ar), cortical thickness (Ct. Th), and cortical bone mineral density (BMD) (Figure 1F). While age-dependent bone loss is extensively documented, age-dependent changes in marrow adiposity is under-reported. Analysis of tibiae stained with osmium tetroxide (Figure 1G) showed a significant expansion of MAT volume (Figure 1H) in elderly vs. mature mice, which paralleled age-related increases in body weight and body fat mass (Figure 1I) and serum leptin levels (Figure 1J). These observed age-related changes in skeletal pathology are consistent with senile osteoporosis.

### 3.2 BM of elderly mice possess an expanded Lin^−^LEPR^+^ SSPC pool

**FIGURE 2:**
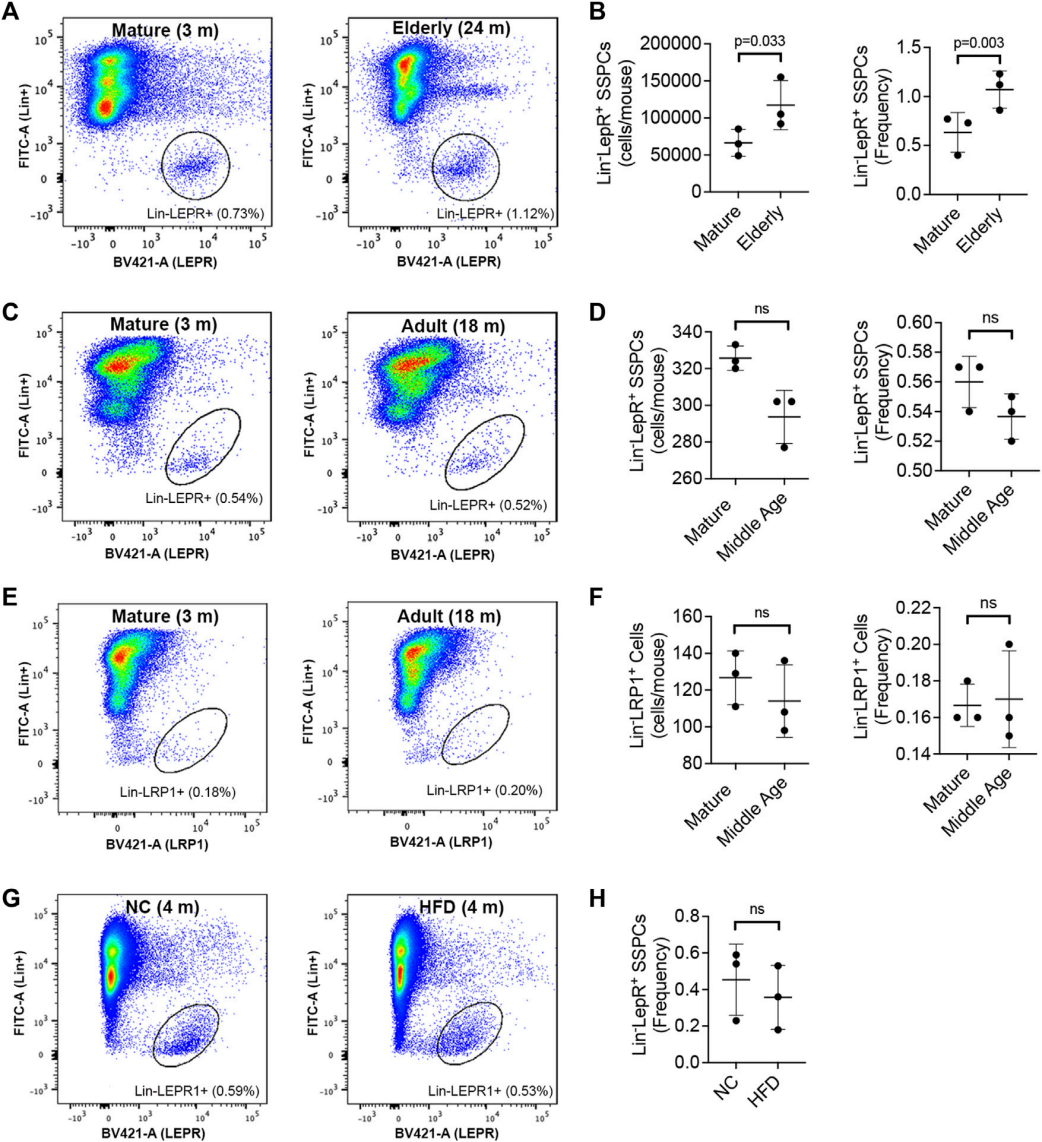
Aging alters the size of the Lin^−^LEPR+ pool in BM. **(A)** Representative flow cytometric dot plots identifying Lin^−^LEPR^+^ cells within the BM of mature (3-month-old) vs. elderly (24-month-old) male mice. **(B)** Bar graphs showing the yield (left) and relative abundance (right) of Lin^−^LEPR^+^ cells based on data from **(A)**. **(C–E)** Representative flow cytometric dot plots of Lin^−^LEPR^+^ **(C)** and Lin^−^LRP1^+^ **(E)** cells in BM of mature (3-month-old) vs. adult (18-month-old) male mice and corresponding bar graphs showing total yields and relative abundance of Lin^−^LEPR^+^ **(D)** and Lin^−^LRP1^+^ **(F)** cells. **(G,H)** Representative flow cytometric dot plots **(G)** and corresponding bar graph **(H)** illustrating the frequency of Lin^−^LEPR^+^ cells in the bone marrow of 3-month-old mice maintained on normal chow (NC) or a high fat diet (HFD) for 100d. All plotted data are mean ± SD from *n* = 3 mice/group and *p*-values by paired Student’s t-test.

To determine if aging alters SSPCs in BM we quantified Lin^−^LEPR^+^ cells in long bones by direct sorting (Figure 2A), which revealed significant increases in both total cell yields and frequency in elderly vs. mature mice (Figure 2B). These results contrast with that from micro-CT analysis, which based on the degree of skeletal involution observed predicts an overall reduction in SSPC numbers. To further validate impacts of aging on the Lin^−^LEPR^+^ SSPC pool, we quantified their frequency in the BM of mature (3-month-old) vs. adult (18-month-old) mice (Figure 2C), which revealed a downward trend consistent with previous reports (Shen et al., 2021) but observed differences were not statistically significant (Figure 2D). Expressed levels of low-density lipoprotein receptor-related protein 1 (LRP1), an endocytic receptor that binds activated forms of A2M, also did not differ significantly in mature vs. adult mice (Figures 2E, F). Since LRP1 was detected in the same cell compartment as LEPR, these data suggest that Lin^−^LEPR^+^ SSPCs may be sensitive to signaling induced by activated A2M via LRP1 binding. Notably, expansion of the Lin^−^LEPR^+^ pool in BM is unique to aging as this population did not significantly differ in the BM of diet induced obese mice (Figures 2G, H), which exhibit similar skeletal pathology as observed in elderly mice (Boregowda et al., 2022).

### 3.3 Aging alters the Lin^−^LEPR^+^ SSPC transcriptome

**FIGURE 3:**
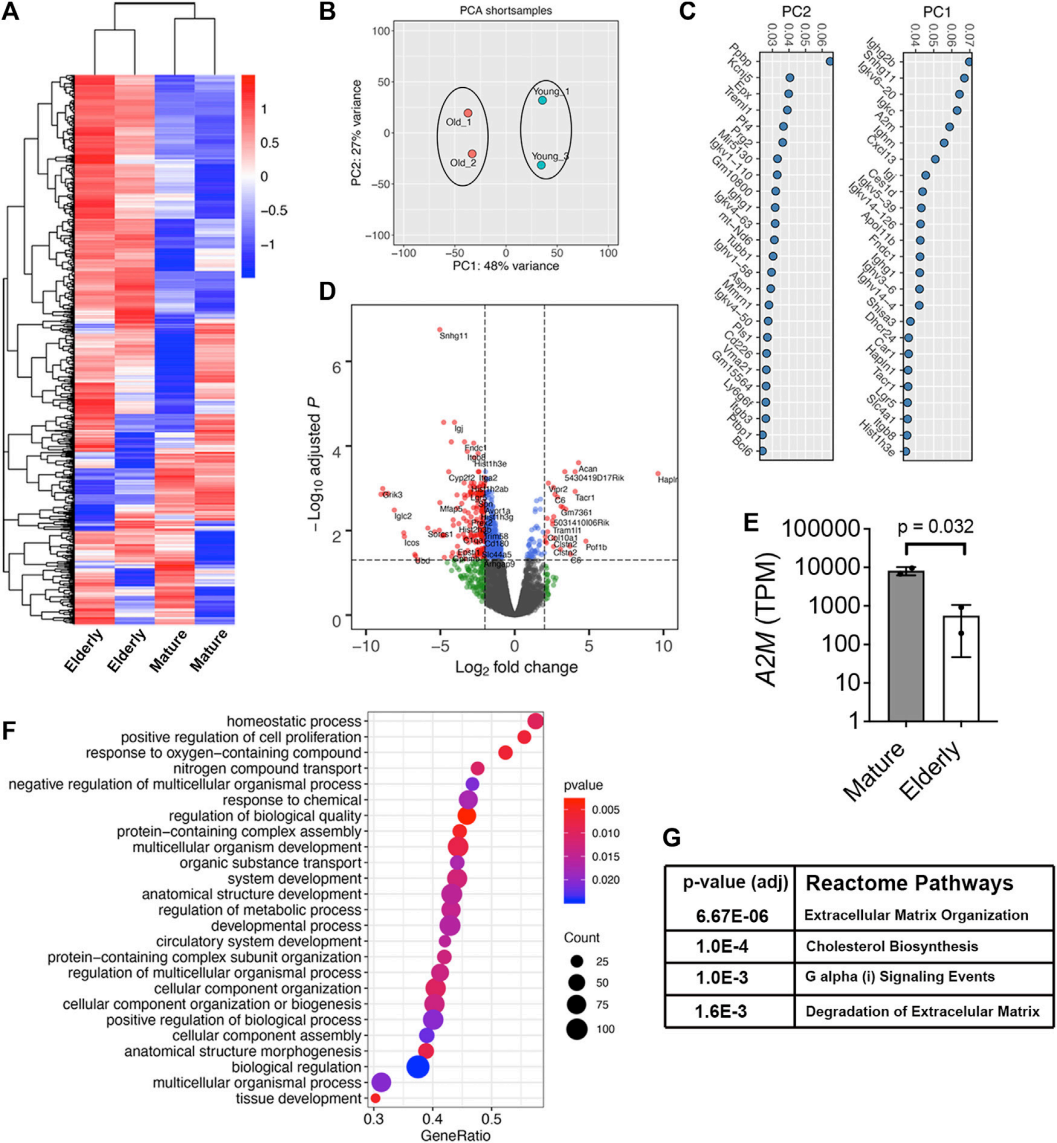
*A2m* is downregulated in Lin^-^LEPR^+^ SSPCs from elderly vs. mature mice. **(A)** Heat map of the top 1000 expressed transcripts in Lin^−^LEPR^+^ SSPCs enriched from the BM of mature (3-month) vs. elderly (24-month) male mice based on RNA-Seq analysis. Colors correspond to per-gene z-score computed across each row. **(B)** PCA analysis of data from **(A)**. **(C)** Gene sets contributing to PC1 and PC2 based on data from **(B)**. **(D)** Volcano plot showing Log_2_ fold-change (FC) values for DEGs and their corresponding *p*-values in Lin^−^LEPR^+^ cells from **(A)**. **(E)** Transcripts per million counts (TPM) of A2M in Lin^−^LEPR^+^ cells from mature vs. elderly mice. Plotted data are mean ± SD and *p*-value by paired Student’s t-test. **(F)** Top gene ontology (GO) terms based on *p*-value identified by GSEA of DEGs in Lin^−^LEPR^+^ cells from **(D)**. **(G)** Most significantly changed Reactome pathways based on DEGs from **(D)**.

We also performed RNA-Seq on freshly isolated Lin^−^LEPR^+^ SSPCs from mature and elderly mice. Hierarchical clustering of the top 1000 expressed transcripts (Figure 3A) and principle-component (PC) analysis (Figure 3B) of these data segregated samples based on donor age. Genes associated with PC1 included *A2m,* transcripts implicated in obesity and energy/lipid metabolism (*Snhg11*, *Dhcr24*, *Ighm*), FGF and WNT signaling (*Shisa3*, *Lrg5*), bone resoprtion (*Car1*), cellular senescence (*Hist1h3e*) and ECM binding/degradation (*Hapln1*, *Itgb8*, *Fndc1*), and transcripts associated with PC2 included genes implicated in innate immunity and inflammatory signaling (*Ppbp*, *Epx*, *Trem1*, *Prg2*, *CD226*), bone mineralization (*Aspn*), cell-matrix adhesion (*Itgb3*) and apoptosis (*Bcl6*) (Figure 3C). This analysis also identified 50 and 286 differentially expressed genes (DEGs) that were up or downregulated, respectively, by >Log2 fold-change (*p* < 0.05) in mature vs. elderly Lin^−^LEPR^+^ SSPCs (Figure 3D). Herein, *A2m* was highly expressed in Lin^−^LEPR^+^ SSPCs from mature vs. elderly mice (Figure 3E) and represented the 5th most highly downregulated transcripts (Log_2_FC = −4.06, *p* = 3.63 × 10^−7^) in elderly mice. Gene set enrichment analysis (GSEA) identified “homostatic process,” “cellular component assembly,” “cellular component organization,” “cellular component biogenesis” and various terms related to multi-cellular organism and system development as the most significant gene ontology (GO) terms mapping to DEGs in elderly vs. mature cells (Figure 3F). Mapping DEGs onto the Reactome pathways identified “extracellular matrix organization,” “degradation of matrix,” and “cholesterol biosynthesis” as the most significantly altered terms in elderly vs. mature cells (Figure 3G). Since cholesterol biosynthesis is elevated in adipogenesis and matrix degradation is a driving force responsible for bone loss, these data are consistent with skeletal pathology observed in elderly mice (Figure 1).

### 3.4 A2M knockdown in human BM-MSCs recapitulates SSPC dysfunction in mice

**FIGURE 4:**
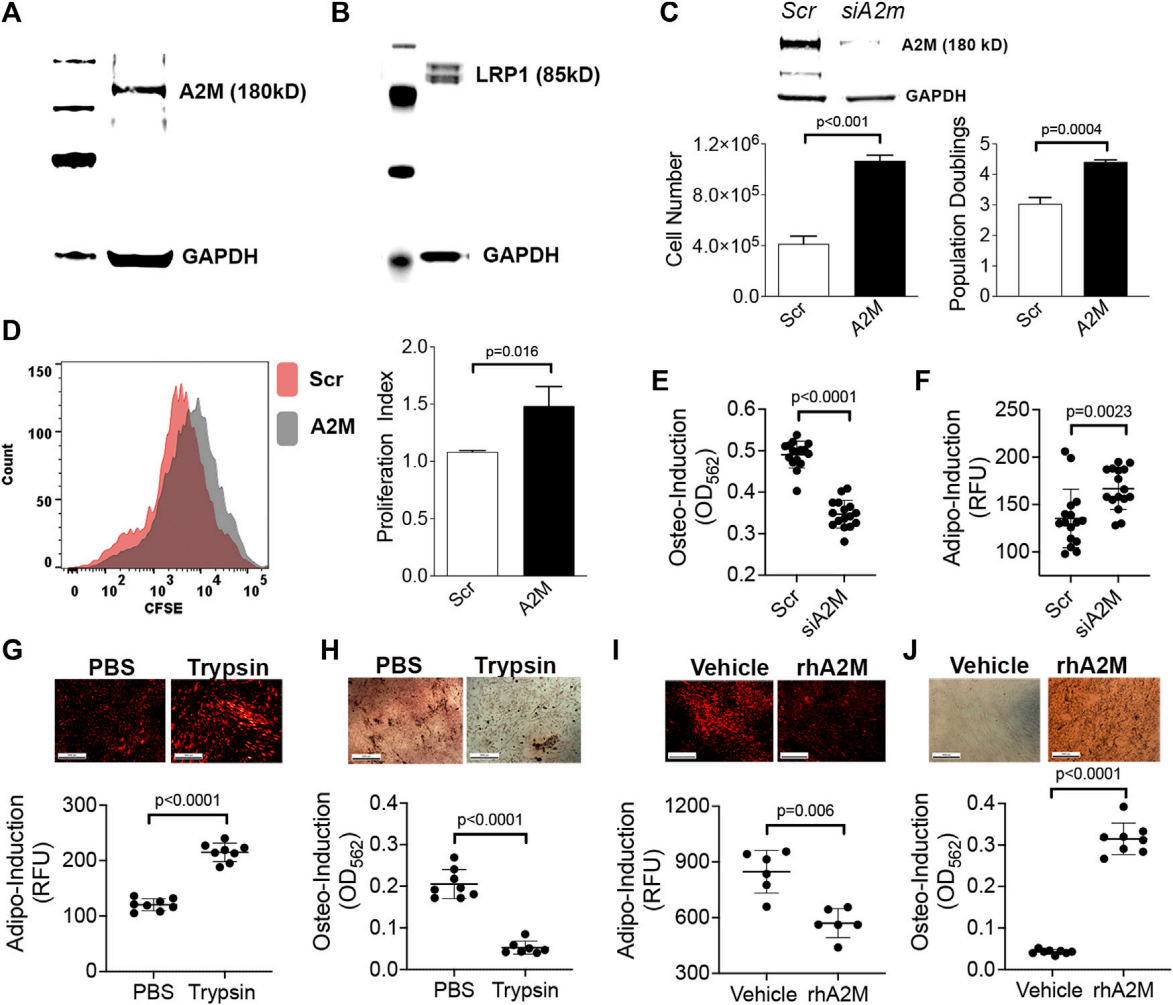
A2M gain and loss-of-function skews lineage bifurcation of human BM-MSCs. **(A,B)** Immuno-blots of BM-MSC cell extracts confirming expression of A2M **(A)** and LRP1 **(B)** protein expression. **(C)** Bar graphs showing cell yield and population doublings of BM-MSCs at 5d post-transfection with a scrambled or *A2m*-specific siRNA. Data are mean ± SD of triplicates and *p*-value by unpaired Student’s t-test. Insert is immuno-blot of cell extracts from scrambled or *A2m*-specific siRNA transfected BM-MSCs. **(D)** Proliferation index (right panel) calculated based on CFSE dilution in BM-MSCs from **(C)** via flow cytometry. Histogram (left panel) shows dilution of CFSE at 5d post-transfection. **(E,F)** Quantification of osteogenic **(E)** and adipogenic **(F)** differentiation of BM-MSCs from **(C)** at 21d and 14d post-induction, respectively. Cell monolayers were stained with AdipoRed™ or Alizarin Red S to quantify fat and mineral deposits, respectively. Plotted data are mean ± SD of replicates (*n* = 8) from two independent experiments and *p* values by unpaired Student’s t-test. **(G–J)** Adipogenic **(G,I)** and osteogenic **(H,J)** differentiation of BM-MSCs following co-culture with trypsin **(G,H)** or rhA2M protein **(I,J)**. Photomicrographs show monolayers stained with AdipoRed™ at 7d post induction **(G,I)** and Alizarin Red S at 5d post induction **(H,J)**. Plotted data are mean ± SD of technical replicates (*n* = 8) and *p*-values by Student’s t-test.

To further interrogate A2M function and extrapolate data from mouse studies to humans, we confirmed that A2M (Figure 4A) and its receptor LRP1 (Figure 4B) are expressed in a well-characterized population of human BM-MSCs by immuno-blotting. We further showed that siRNA-mediated silencing of A2M significantly stimulated proliferation of these BM-MSCs based on counting (Figure 4C) and flow cytometric analysis of CSFE labeled cells (Figure 4D). A2M silencing also significantly inhibited the ability of BM-MSCs to undergo stimulus-driven osteogenic differentiation (Figure 4E) but increased their capacity for stimulus driven adipogenic differentiation (Figure 4F), which is consistent with skeletal pathology observed in elderly mice. Next, we cultured BM-MSCs stimulated to undergo adipogenic or osteogenic differentiation in the presence of trypsin or rhA2M to mimic the BM microenvironment of elderly and mature mice, respectively. Under conditions of increased proteolysis BM-MSCs exhibited increased adipogenesis (Figure 4G) and decreased osteogenesis (Figure 4H) while in the presence of rhA2M, they exhibited decreased adipogenesis (Figure 4I) and increased osteogenesis (Figure 4J). Together, these data indicate that increased proteolytic remodeling of the ECM skews lineage bifurcation of BM-MSCs toward fat lineages at the expense of bone.

**FIGURE 5:**
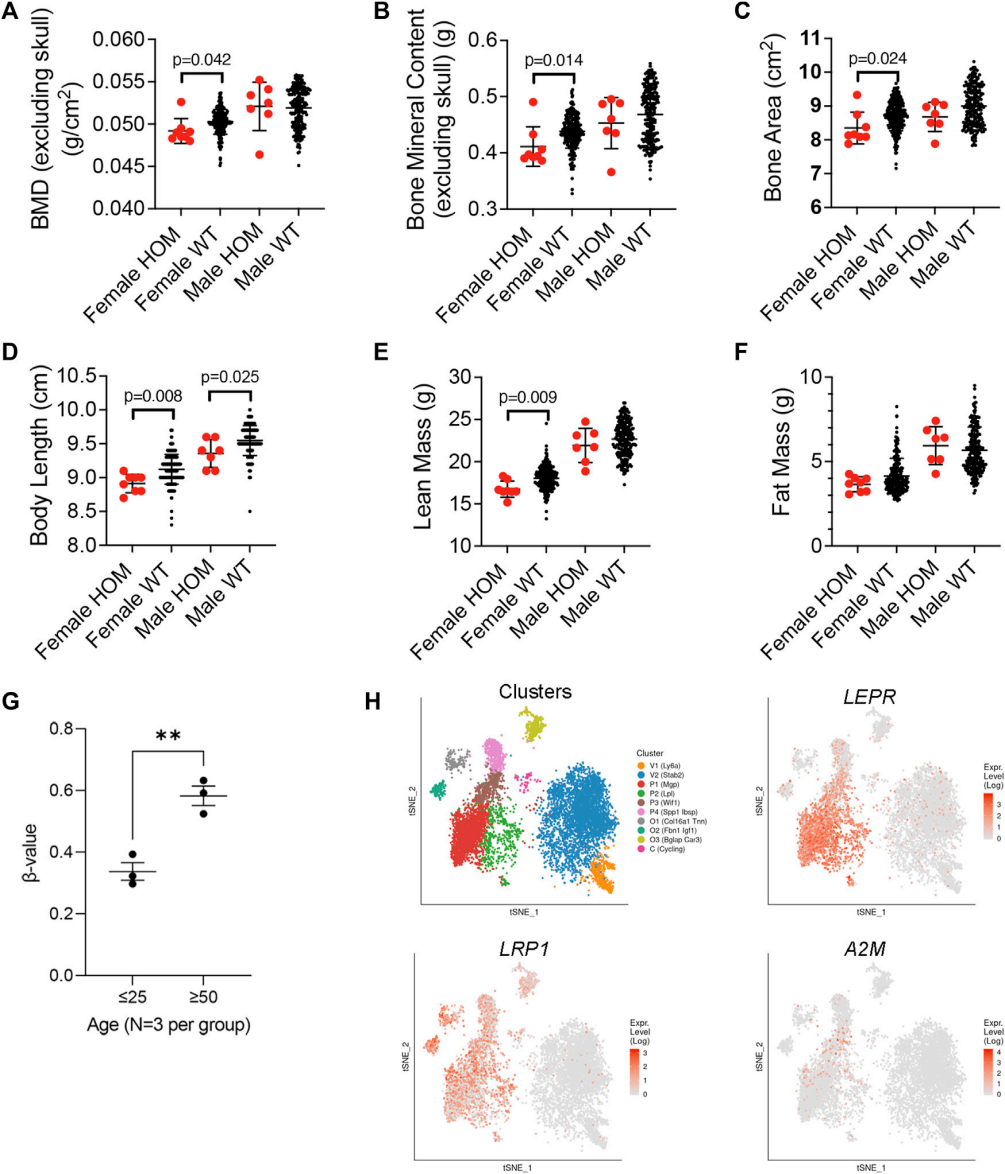
Expression profiling of *A2m* in BM and phenotypic impacts of its loss of function in mice. **(A–F)** Body composition phenotypic assays of BMD **(A)**, bone mineral content **(B)**, bone area **(C)**, body length **(D)**, lean **(E)** and fat mass **(F)** in 8 female and 7 male 13-week-old homozygous mutant (HOM) A2m^tm1b(NCOM)Mfgc^ mice and 226 female and 209 male wild type mice. Data are from the International Mouse Phenotypic Consortium. *p*-values determined by unpaired Student’s t-test. **(G)** Methylation status of 27,578 unique CpG sites in human BM-MSCs from young (≤25 years old) vs. elderly (≥50 years old) donors (GSE17448) and used to quantify the extent of A2M promoter methylation plotted as the intensity ratio between methylated and unmethylated alleles (β-values). Values range from 0 to 1 with 0 being unmethylated. **p < 0.01 by unpaired Student’s t-test. **(H)** Color coded visualization of tSNE analysis of single cell RNA-Seq of bone marrow niche cells. (*n* = 17,347 cells). P1, P2, P3, and P4 are subpopulation clusters of LEPR+ SSCs wherein P1, P3, P4 represent osteogenic skewed populations, and P2 represents adipogenic-skewed cells. Expression landscape of *Lepr*, *Lrp1*, and *A2m* are shown. O1, O2, and O3 represent osteoblast and V1 and V2 endothelial cells. Data and visualization taken from publicly available online source of single cell RNA seq analysis of the bone marrow niche cells called niche explorer (https://compbio.nyumc.org/niche/).

To further confirm a role for A2M as a disease modifying protein in senile osteoporosis, we compared data on body and skeletal composition of wild type and *A2m* homozygous mutant (HOM) mice from the International Mouse Phenotypic Consortium (https://www.mousephenotype.org/). Analysis of the body composition of 13-week-old HOM mice revealed significantly lower BMD (Figure 5A), bone mineral content (Figure 5B), and bone area (Figure 5C) compared to wild type (WT) female mice. Although these metrics did not differ significantly between wild type and HOM male mice, a similar trend toward lower bone area and mineral content was evident. A2M HOM mice of both sexes also exhibited significantly shorter body length (Figure 5D), an indirect measure of bone formation, than their WT counterparts, while lean body mass was significantly lower in female HOM vs. WT mice (Figure 5E) and fat mass trended higher in female HOM vs. WT mice (Figure 5F). Together, these data indicate that knockout of *A2m* yields observable skeletal defects in young mature mice. Since *A2m* expression is downregulated in response to aging in SSPCs, we inspected data published by Bork et al. (2010), comparing DNA methylation chagnes in 27,578 unique CpG sites in human BM-MSCs from young (≤25 years old) vs. elderly (≥50 years old) donors. This analysis revealed a significant increase in DNA methylation at the *A2m* promoter (Figure 5G) suggesting that epigenetic mechansims may contribute to downregulation of A2M in BM-MSCs in response to aging. Lastly, we examined *A2M* expression within the T-distributed Stochastic Neighbor Embedding (tSNE) landscape generated by Tikhonova et al. (2019). This analysis indicated that A2M expression is enriched in *Mgp* and *Wif1* expressing subpopulations of Lin-/LEPR+ SSPCs that are skewed toward osteogenesis compared to *Lpl* expressing cells that are skewed toward the adipogenic lineage (Figure 5H). Moreover, A2M appears to be highly enriched in the P3 and to a lesser extent P1 subpopulations where P3 is designated as a key bifurcation point on the trajectory from osteogenic and adipogenic precursors. Therefore, A2M may be important in balancing lineage commitment of P3 cells toward bone and fat, and its downregulation in response to aging may skew this balance toward adipogenesis at the expense of osteogenesis. Importantly, *A2M* expression overlaps with *LRP1*, which is consistent with data from BM-MSCs, and is restricted to a smaller subset of BM cells as compared to *LEPR.*

## 4 Discussion

A large body of literature exists describing how alterations to niche architecture and function contribute to exhaustion of various tissue-specific stem/progenitor populations in response to injury and/or aging (Koester et al., 2021; Pohl et al., 2021; Zhang et al., 2021; Ho and Takizawa, 2022; Wu et al., 2023). Herein, we identify *A2m* as a highly downregulated transcript in BM resident Lin^−^LEPR^+^ SSPCs from elderly vs. mature mice and show that silencing its expression in human BM-MSCs recapitulates key aspects of age-induced SSPC dysfunction *in vivo.* These findings, together with phenotypic data from HOM *A2m* mice, identify A2M as a putative disease modifying protein in senile osteoporosis. A2M functions as a pan-protease inhibitor produced by the liver, is normally found at high concentrations in the blood, and its expression is inversely correlated with age in humans (Birkenmeier et al., 2003) Recently, A2M purified from blood was approved by the FDA for treating osteoarthritis via direct injection into joints wherein it prevents joint damage by inactivating resident matrix degrading proteases (Wang et al., 2014). Importantly, A2M exists in blood as a 720 kD tetramer and is not present in large quantities in synovial fluid but can be administered to joints by local injection. These data indicate that A2M does not readily exit the blood stream and therefore has limited bioavailability in tissues. Consequently, A2M secreted by SSPCs may function locally in niche maintenance by modulating protease dependent remodeling of the ECM. Activated forms of A2M also bind LRP1 (CD91), which functions as an endocytic receptor but also plays roles in cell adhesion, proliferation, migration, lipid metabolism and other processes (Lillis et al., 2005; Etique et al., 2013). Therefore, A2M may also function in an autocrine manner to impact these key processes in SSPCs. Lastly, A2M is also implicated in tissue inflammation (Burgess et al., 2008) via binding of various cytokines (IL1, IL6, TNF, TGF-β2) and may preserve SSPC function by reducing levels of inflammatory cytokines induced in response to age-induced oxidative stress.

Our data indicate that A2M downregulation skews BM-MSCs toward adipogenesis while promoting increased cell proliferation. These findings are unique in that all genes studied in our laboratory including TWIST1 (Boregowda et al., 2016), TWIST2 (Lai et al., 2011), P53 (Boregowda et al., 2018), and IP6K1 (Boregowda et al., 2017) that modulate BM-MSC growth and differentiation produce a consistent phenotype; those that positively regulate cell proliferation also promote osteogenesis at the expense of adipogenesis. These results are consistent with the fact that adipogenesis is preceded by cell growth arrest in BM-MSCs (Zhang et al., 2020). Therefore, A2M is an exception to this rule since A2M inhibition stimulates growth and adipogenesis. This phenomenon is consistent with data showing downregulation of A2M drives expansion of the Lin^−^LEPR^+^ SSPC pool and skews their bifurcation toward adipogenesis, which reconciles observed skeletal pathology in mice and humans afflicted with senile osteoporosis. However, whether this results from cell-autonomous or systemic impacts of A2M on SSPC function is indeterminate and requires further study.

## Data Availability

The datasets presented in this study can be found in online repositories. The names of the repository/repositories and accession number(s) can be found below: GEO database (GSE243475).
