# Supplementary material for: Novel role for alpha-2-macroglobulin (A2M) as a disease modifying protein in senile osteoporosis
Source: Front Cell Dev Biol. 2023 Oct 30;11:1294438. doi: 10.3389/fcell.2023.1294438 (PMC10642388; doi:10.3389/fcell.2023.1294438)
Supplement: Supplementary file 1 [file Presentation1.zip › Table 1 (6).DOCX]

Supplementary Material

## 1. Supplementary Figures

**Supplementary Figure 1.** A & B, Uncropped immuno-blots (left panels) of BM-MSC cell extracts probed with the anti-A2M(A) and anti-LRP1 (B) antibodies followed by Alexa Fluor 680 xxxxxxx (red) and IRDye 800 xxxxxxxxxx (green). Right panel is black & white image of fluorescent blot and red square is aspect of blot shown in Figure 4. C, Uncropped immuno-blot of cell extracts from BM-MSCs transfected with a scrambled or A2M-specific siRNA and probed with anti-A2M and anti-GAPDH as in (A). Both colors were imaged in a single scan.

**
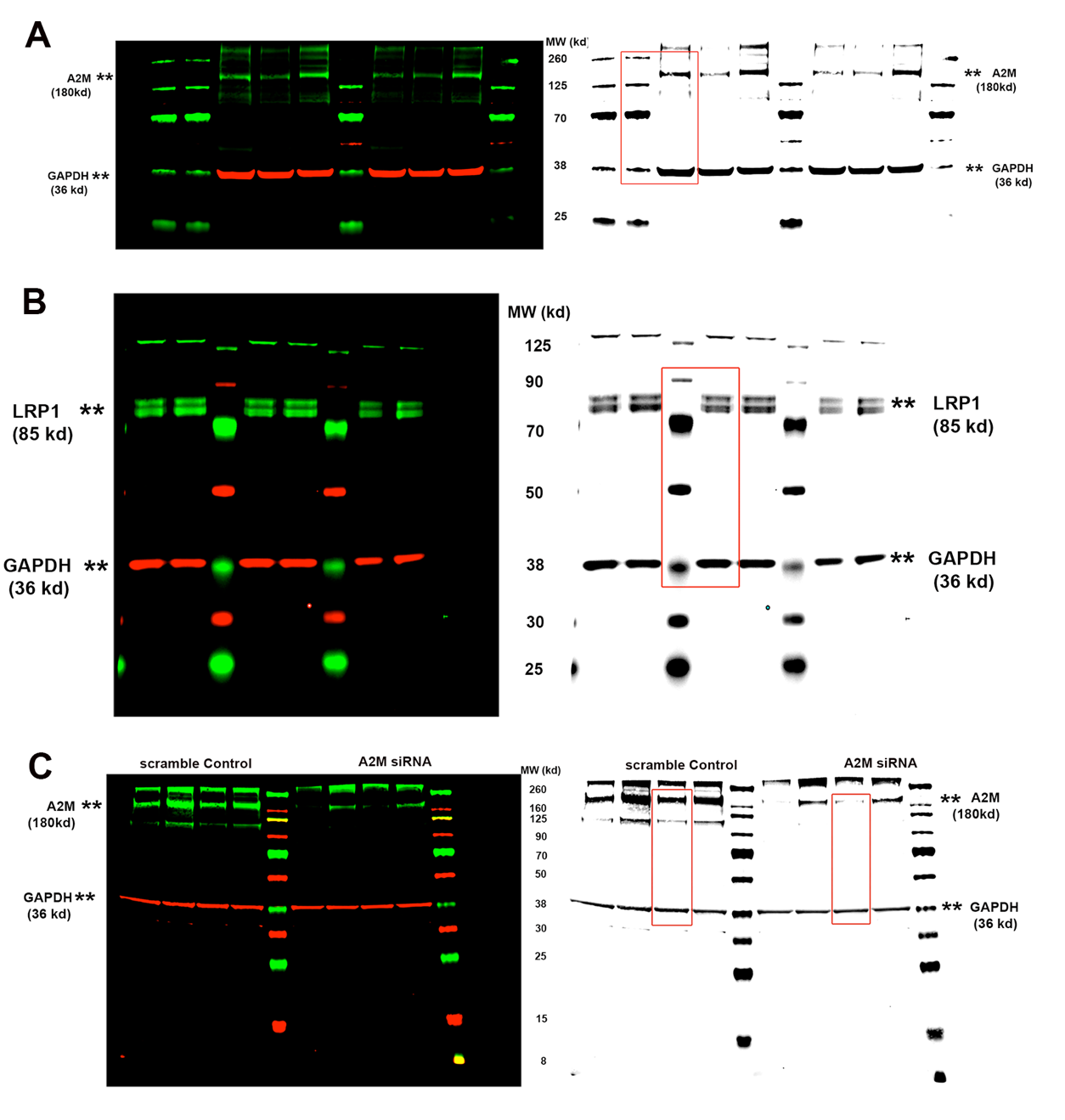
**
